# Takayasu Arteritis: A Diagnosis Made Using a Multidisciplinary Approach

**DOI:** 10.7759/cureus.2781

**Published:** 2018-06-11

**Authors:** Quratulain Fatima Masood, Anika Naeem, Syed Maaz Tariq, Ali Asad, Sara Habib

**Affiliations:** 1 Surgery, National University of Science and Technology, Rawalpindi, PAK; 2 Graduate, Allama Iqbal Medical College, Lahore, Pakistan, Lahore, PAK; 3 Department of Medicine, Jinnah Sindh Medical University (SMC), Karachi, PAK; 4 Pediatric Surgery, University of Health Sciences, Lahore, Lahore, PAK; 5 Neuro-Critical Care, Thomas Jefferson University, Philadelphia, USA

**Keywords:** arteritis, takayasu, stenosis

## Abstract

Takayasu arteritis is an idiopathic chronic vasculitis that involves large blood vessels, including the aorta and its main branches. This disease presents typically as ischemia or aneurysms that could be prevented by timely diagnosis and vigilant management. We present here the case of a 19-year-old male who presented with a history of visual disturbance, chest pain, dizziness, and a feeble pulse.

## Introduction

Takayasu is a giant cell arteritis, which is predominant in young women. Pathology suggests an inflammatory etiology, with a rare incidence of 150 cases annually occurring in Japan or less than three cases per million annually occurring in the United States and Europe [1]. Diagnosis can be made by computed tomography (CT) angiography, especially when the vascular manifestation predominates the presentation. Ultrasonography is used for carotid assessment, while 18F-fluorodeoxyglucose positron emission tomography (18F-FDG-PET) is convenient for diagnosing patients with no vascular signs or symptoms. Inflammatory markers and auto-antibodies tend to be nonspecific while diagnosing Takayasu arteritis.

## Case presentation

**Figure 1:**
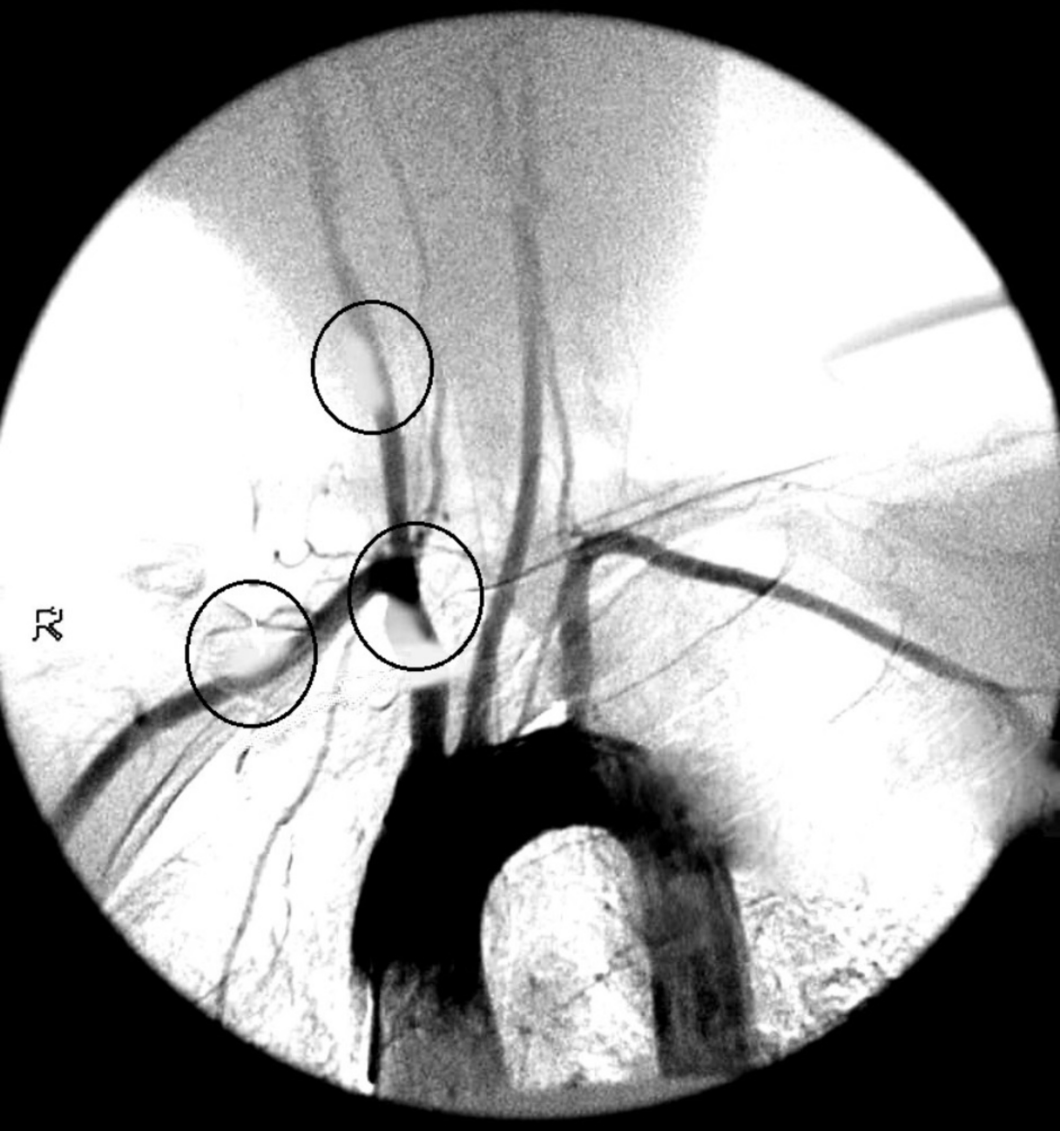
Computed tomography angiography showing occlusions stenoses.

The patient had an episode of left-sided paralysis a year ago, and he presented with complaints of dizziness, chest pain radiating down the left arm and back, tachycardia, palpitations, dizziness, night sweats, and dyspnea. Visual disturbances included myopia and hyperopia with a bilateral temporal headache. Physical findings on examination included diaphoresis, with marked pallor, cold and sweaty hands and face. The pulse was absent bilaterally in both the upper limbs. Bruits were heard on the carotids. An itchy and erythematous rash was present on the upper torso. An electrocardiogram (EKG) was unremarkable. A Doppler echocardiographic study was performed, which concluded that the patient had Grade 1 diastolic dysfunction. A CT angiography showed stenoses of brachiocephalic trunk, right subclavian and common carotid artery (all shown in Figure 1).

Our case fulfilled the obligatory criteria of diagnosis. Patient management with methotrexate and steroids failed. The addition of leflunomide resulted in no clinical progress. Tocilizumab helped in the crude resolution of symptoms only. Percutaneous transluminal angioplasty with stenting of the stenosis under sedation was performed to avoid compromising the arteries. Perioperative inflammation was controlled using steroids and tocilizumab. To avoid relapse, tocilizumab was continued postoperatively while tapering down steroids.

Simultaneous carotid artery stenting can increase the risk of cerebral hyperperfusion syndrome, and it can cause severe bradycardia or hypotension related to bilateral baroreceptor irritation; therefore, frequent monitoring of blood pressure [2] and neurologic symptoms was done.

## Discussion

Takayasu arteritis is a clinically vague condition, which is often difficult to diagnose. It presents with constitutional symptoms such as arthralgia, hypertension, dizziness, visual disturbances, carotidynia, skin lesions, and neurologic, respiratory and gastrointestinal symptoms. Treatment often commences with steroids. However, cases where disease commences prior to 20 years of age are refractory to medical intervention and they require a vascular surgical intervention. Refractory disease means even after standard treatment, the disease worsens only showing minimal improvement.

Usually, steroids show a good response, but relapse occurs in 72% of the patients on tapering steroids. Those kept on long-term steroid therapy face various dangerous side effects of steroid use. Steroids are absolutely futile if inflammation leads to scarring around a vessel. Tapering down steroids results in relapse, which could be managed by administering methotrexate and leflunomide but unfortunately their anti-inflammatory properties are not strong enough to counter the scarring of vessel. At this point, angioplasty with stenting can alleviate the symptoms. Percutaneous transluminal angioplasty with stenting should be reserved for short lesions that are refractory to medication, but the odds of restenosis occurring are high if inflammation continues after vascular intervention. The use of steroids and tocilizumab preoperatively, and the use of tocilizumab postoperatively are a marvelous combination that not only control inflammation but also reduce the incidence of postoperative complications like stroke [3] restenosis, aneurysm, thrombosis, embolism, hypertension, neurologic impairment, and death [4]. Tocilizumab is an antibody, a biological agent that binds interleukin 6 receptor and hinders its pro-inflammatory effect [5]. Hence, tocilizumab aids in the long-term patency of the vascular intervention, effectively controlling the patient's condition, improving the rate of remission, ensuring relapse-free survival, and arresting damage progression.

With vascular intervention, symptoms resolve in 93% of the patients but if restenosis occurs, symptoms worse than the initial presentation can occur. Our patient was followed with multiple complementary diagnostic techniques, and tocilizumab was administered immediately after stenting to avoid complications, which were highly possible and unpredictable even after vascular intervention. Vascular intervention provides imminent relief to patients with refractory Takayasu arteritis, but surgery-associated complications and active disease conversely decreased the survival of patients. Administering tocilizumab decreases inflammation which, if left untreated could restenose the vessel, hence reproduce the symptoms.

Our case shows that the strength of treatment for refractory Takayasu arteritis lies in the use of a combined treatment, including vascular intervention, appropriate medication, and precise follow-up after discharge. Where surgery provides imminent relief from refractory symptoms, relapse and postoperative complications can only be averted using medications.

## Conclusions

Takayasu arteritis is a granulomatous vasculitis typically prevalent in young women of Asian origin. Symptoms comprise neurologic manifestations, pulselessness, and cardiac manifestations. Imaging techniques help in identifying the vessels involved. Inflammatory markers are nonspecific. Treatment modalities involve steroids and disease modifying agents. Scarring stemming from inflammation can curb the efficacy of the medication. If symptoms do not abate, then angioplasty with stenting is an alternative. This approach resolves the symptoms but complications can have fatal consequences. These consequences can be fended off using a strong anti-inflammatory combination which can ensure good prognosis of surgical interventions along with combating postoperative complications. Tocilizumab and steroid combination has worked wonders to serve our purpose. 
